# MRI features of Stewart-Treves syndrome: a case series and systematic review

**DOI:** 10.1007/s11604-025-01744-2

**Published:** 2025-02-05

**Authors:** Nobuo Kashiwagi, Atsushi Kawata, Mio Sakai, Hiroto Takahashi, Tomoko Hyodo, Hayato Kaida, Ken-ichi Yoshida, Satoshi Nojima, Satoshi Takenaka, Kazunari Ishii, Katsuyuki Nakanishi, Noriyuki Tomiyama

**Affiliations:** 1 Department of Diagnostic and Interventional Radiology, Osaka International Cancer Institute, 3-1-69 Otemae, Chuo-ku Osaka, 541-8567 Japan; 2 Department of Radiology, Osaka University Graduate School of Medicine, 2-2 Yamadaoka, Suita, 565-0871 Japan; 3 Department of Radiology, Faculty of Medicine, Kindai University, 377-2 Ohno-Higashi, Osaka-Sayama, 589-8511 Japan; 4 Department of Diagnostic Pathology and Cytology, Osaka International Cancer Institute, Chuo-ku Osaka, Japan; 5 Department of Pathology, Osaka University Graduate School of Medicine, Suita, Japan; 6 Musculoskeletal Oncology Service, Osaka International Cancer Institute, Chuo-ku Osaka, Japan

**Keywords:** Stewart-Treves syndrome, Angiosarcoma, MRI, Systematic review

## Abstract

**Purpose:**

To comprehensively summarize the magnetic resonance imaging (MRI) features of angiosarcomas presenting as Stewart-Treves syndrome (STS) through a retrospective case series and systematic review of previous publications.

**Materials and methods:**

We identified five patients with STS from our institutional database and 25 patients with STS from 15 publications through a systemic review. We reviewed the MR features of 30 patients with STS, including five males and 25 females with a mean age of 59.5 years.

**Results:**

The tumors most commonly involved both the cutis and subcutis (17/25, 68.0%); the remaining tumors were limited to the cutis or subcutis. Multiple tumors were observed in more than half of the cases (16/27, 59.3%), and most of the tumors had poorly defined margins (26/28, 92.9%). The most common signal intensities of the tumors on T1-weighted images were intermediate (16/19, 84.2%), and the remainder were a mixture of intermediate and high, with a predominance of intermediate signal intensity. The signal intensities of the tumors on T2-weighted images were intermediate in seven cases (7/13, 53.8%), a mixture of intermediate and high in five cases (5/13, 38.5%), and a mixture of intermediate and low in one case (1/13, 7.7%). Available diffusion-weighted images from four institutional cases showed restricted diffusion of the tumors with mean apparent diffusion coefficient (ADC) values ranging from 0.77 × 10^−3^ mm^2^/s to 0.96 × 10^−3^ mm^2^/s.

**Conclusion:**

Typical MRI features of angiosarcomas in STS were superficially located in multiple masses with ill-defined margins. Internal signal intensity was intermediate on T1-weighted images, and intermediate or a mixture of intermediate and high on T2-weighted images. ADC values obtained from the limited number of cases were low.

## Introduction

Angiosarcomas are rare but aggressive tumors with high rates of local recurrence, metastasis, and tumor-related deaths [1, 2]. Because they originate from lymphatic or vascular endothelial cells, they can occur in any organ or tissue, either as primary or secondary angiosarcomas [1–3].

Regarding secondary angiosarcomas, the differences from primary angiosarcomas in pathologic phenotype and prognosis are not clear [1, 3, 4] and the two most common risk factors are post-irradiation and long-standing lymphedema, the latter known as Stewart-Treves syndrome (STS) [5]. Chronic lymphedema, which leads to local immunosuppression, can be the etiology of various cutaneous malignancies, including angiosarcoma, melanoma, Kaposi sarcoma, basal cell carcinoma, and squamous cell carcinoma. Among these malignancies, angiosarcoma is the most common, accounting for approximately 90%, and has a worse prognosis than other malignancies, with a reported median survival of 22 months [6].

Therefore, given the rising prevalence of lymphedema, the need for awareness and early diagnosis of STS has been described [6, 7], however, there is a lack of a comprehensive understanding of the imaging features of STS. The purpose of this study was to summarize the magnetic resonance imaging (MRI) features of STS, including diffusion-weighted (DW) images, through a retrospective case study and a systematic review.

## Materials and methods

### Institutional retrospective review

The present study was approved by our institutional review board and the need for informed consent was waived because of the retrospective nature of the study. By retrospectively reviewing the medical records of three cancer referral centers in Japan between January 2010 and December 2023, 208 patients with angiosarcoma or suspected angiosarcoma were identified. After reviewing the individual electronic medical records, eight patients with STS were identified according to the following criteria: (1) histologic diagnosis of angiosarcoma and (2) pre-existing chronic edema at the site of angiosarcoma development. After excluding cases with no available MRI, five patients were finally included.

### Systematic review

**Fig. 1:**
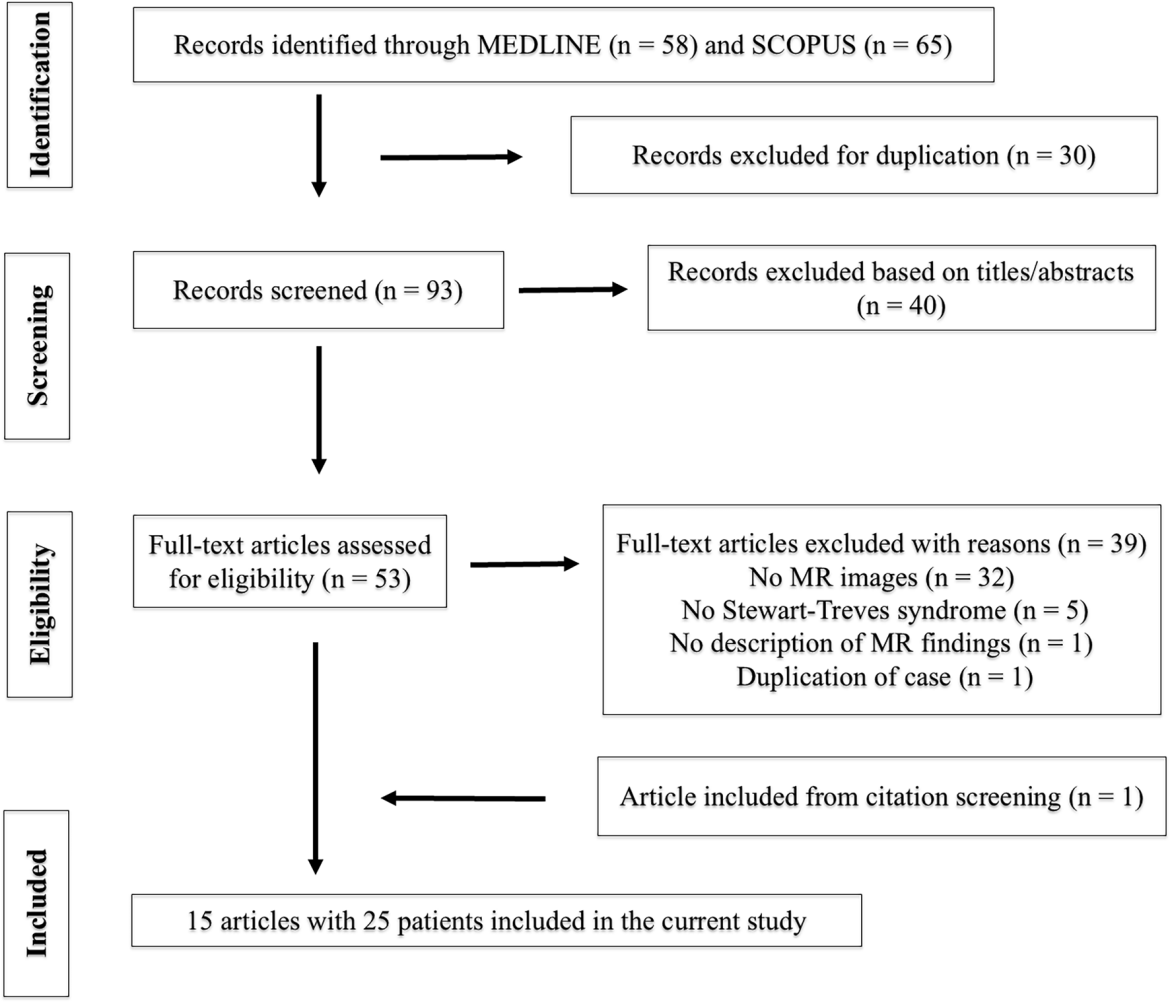
Overview of the article selection process based on the Preferred Reporting Items for Systematic Reviews and Meta-Analyses 2020 Guidelines

We conducted a systematic review in accordance with the Preferred Reporting Items for Systematic Reviews and Meta-Analyses 2020 Guidelines [8], as shown in Fig. 1. The PubMed and Scopus databases were searched for studies published between January 1, 1990, and December 31, 2023. Keyword search terms were as follows: (“Stewart-Treves syndrome”) OR (“angiosarcoma lymphedema”) AND (“MRI”) OR (“magnetic resonance”) OR (“imaging”) OR (“radiology”).

Two reviewers (NK and MS) independently screened the titles and abstracts of the retrieved reports and assessed full texts for eligibility. The eligibility criteria for the identified articles were as follows: (1) studies with an original case of STS; (2) studies providing magnetic resonance (MR) images with a description of their findings; and (3) studies presented in English. The exclusion criteria were cases (1) in which individual patient data were unavailable; (2) in which individual detailed MRI findings could not be extracted (i.e., only aggregated data were reported); and (3) presented in other types of records, including books and conference proceedings, without a complete, peer-reviewed publication. Disparities between the two reviewers were resolved through discussion until a consensus was reached. For potential inclusion of further articles, a citation search of the retrieved eligible articles was performed.

### Extraction of clinical data

By reviewing the individual electronic medical records of five institutional cases and literature of 15 cases from the systematic review, baseline clinical features including age, sex, cause of lymphedema, duration of lymphedema, presenting symptoms, tumor sites, treatments, recurrence sites, and outcomes were extracted.

### Evaluation of MR images

Two board-certified radiologists with 22 and 21 years of experience (MS and HT, respectively) evaluated all MR images of the institutional and reported cases obtained from the systematic review. Differences between the two readers were resolved by a third radiologist with 28 years of experience (NK), whose reading was considered the final result.

The evaluated items included tumor depth (cutis, subcutis, or deep); outer protrusion from the skin surface (present or absent); the number of tumors (single or multiple); tumor shape (amorphous/diffuse, multinodular confluent, or round/oval); margin characteristics (ill-defined or well-defined); signal intensities on T1-weighted images, T2-weighted images, and fat-suppressed T2-weighted images; enhancement patterns (homogeneous, heterogeneous, or ring enhancement); and apparent diffusion coefficient (ADC) values on DW images, which were only available for our institutional cases. In the evaluation of shape, margin characteristics, signal intensities, and ADC values, the largest lesion was observed when multiple tumors were present.

**Fig. 2:**
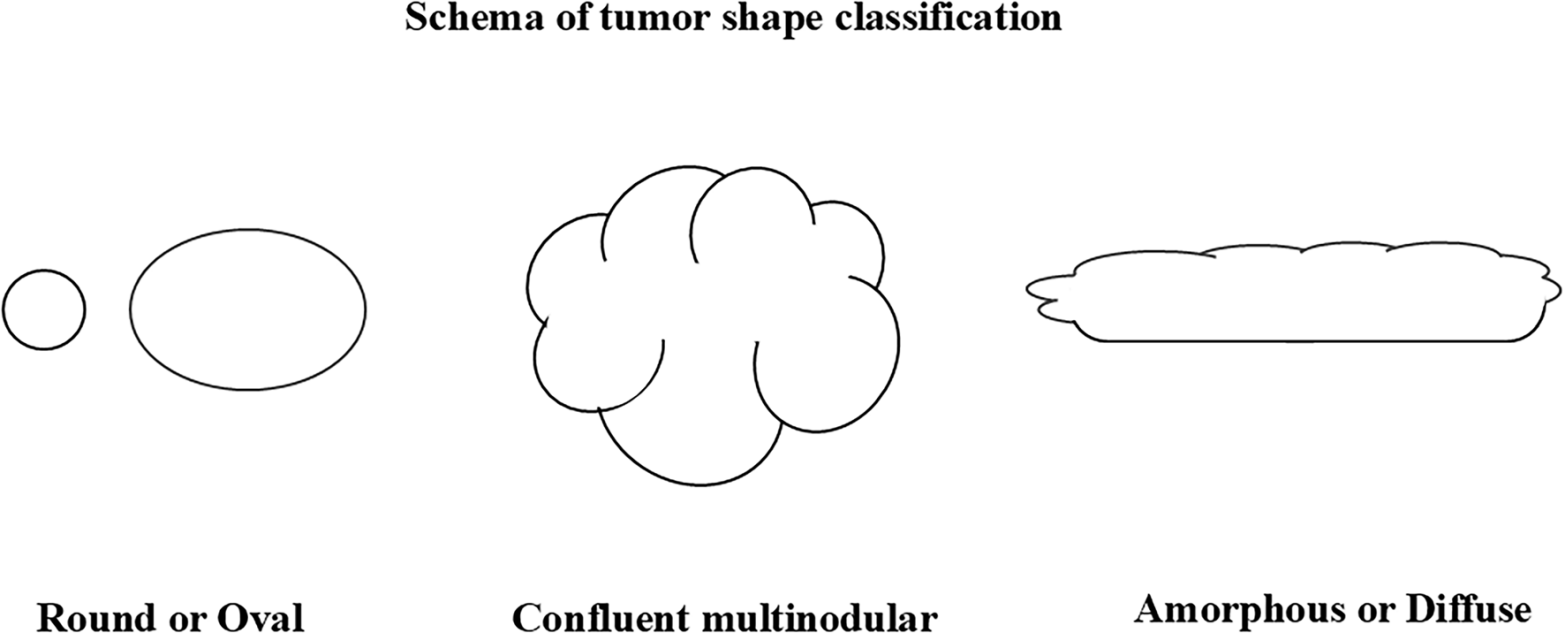
Schema of tumor shape classification. Multinodular confluent is defined as having the shape of multiple small nodules fused together to resemble grape clusters. Amorphous or diffuse is defined when the tumor does not have a clearly defined shape or form

Tumor depth was defined as subcutis if the tumor was located in the subcutaneous fat, and deep if it crossed the peripheral deep fascia [9, 10]. Tumor shape was defined as multinodular confluent when the multiple small nodules fused together to resemble grape clusters, and amorphous or diffuse when the tumor had no clearly defined shape or form (Fig. 2). The signal intensities on T1- and T2-weighted images were classified as high if the signal intensity was equal to or higher than that of fat; intermediate if the signal intensity was lower than that of fat but equal to or higher than that of normal muscle; and low if the signal intensity was lower than that of normal muscle. On fat-suppressed T2-weighted images, high signal intensity was defined as a signal intensity equal to or higher than that of concomitant subcutaneous edema, intermediate signal intensity was defined as lower than that of edema but equal to or higher than that of muscle, and low signal intensity was defined as lower than that of muscle. For the ADC values, the first two radiologists measured the mean ADC (ADC_mean_) values within the region of interest (ROI) on the ADC maps. The ROIs were determined as widely as possible, while excluding the cystic portion, by referring to morphological information including T1-weighted, T2-weighted, and post-contrast T1-weighted images. The average of the measurements by the two radiologists was used as the final value.

For the four institutional cases that were available for pathologic specimens, a third radiologist and a pathologist (KY) correlated the MR images and pathology.

## Results

### Institutional cases series

The clinical features of the five patients are summarized in Table 1. The patients included five females ranging in age from 43 to 77 years. Lymphedema was caused by surgical intervention for uterine cervical cancer or ovarian cancer in three patients, idiopathic in one, and disuse edema following an ischemic stroke in one. The duration of lymphedema ranged from 8 to 18 years. The presenting symptoms included a palpable mass in three patients, painful skin exanthesis in two, and bleeding in one. Tumors were located in the lower leg in three patients and in the lower abdominal wall in two. Histologic diagnosis was made by biopsy in two patients and by surgical resection in three patients. Of the former two patients, one received palliative therapy and the other received chemoradiotherapy. Of the latter three patients, two had positive pathologic margins and were subsequently treated with adjuvant chemotherapy. Of the four patients treated curatively, three relapsed; two patients developed local recurrence, one developed systemic lymph node metastases and one developed lung metastases. In the final outcome, three patients died of the disease between 6 and 23 months, one is alive with disease after 60 months of follow-up, and one was alive without disease after 481 months of follow-up.

**Table 1:**
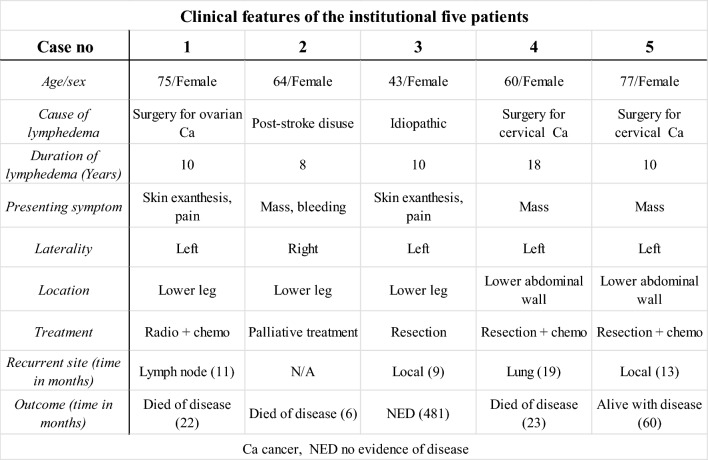
Clinical features of the institutional five patients

**Fig. 3:**
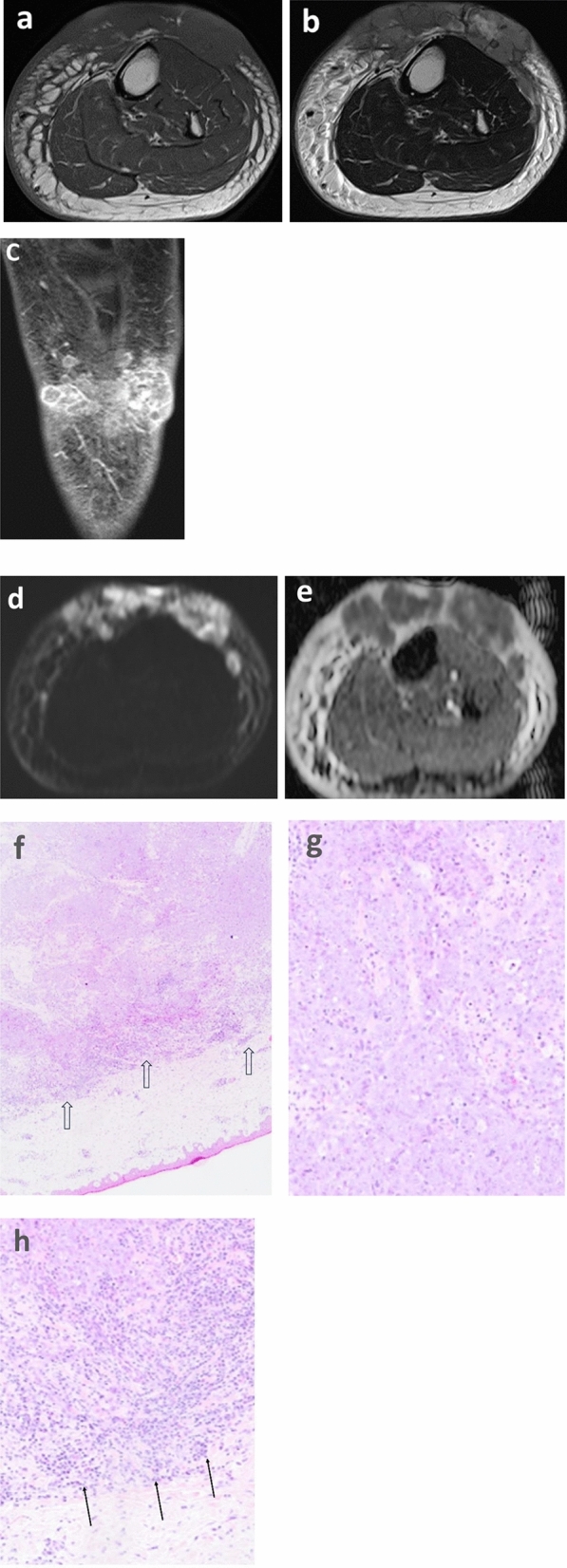
A 75-year-old female, with a 10-year history of lower extremity lymphedema following hysterophenectomy for ovarian cancer presented with painful dark red exanthesis of the left lower leg (case 1). **a** Axial T1-weighted image shows multiple ill-defined masses with intermediate signal intensities. **b** On axial T2-weighted image, the signal intensities of the masses are mainly intermediate and partly high. **c** Coronal contrast-enhanced fat-suppressed T1-weighted image shows the confluent multinodular shapes of the masses. **d** and **e** DW image and ADC map show hyperintense masses with low ADC values (ADC_mean_ = 0.96 × 10^−3^ mm^2^ /s) on the ADC map. **f** Microscopy of a lower-power field shows a predominant distribution of a solid sheet of tumor cells and a convoluted border between the tumor and subcutaneous connective tissue without capsular formation (double-lined arrows). The former appears to contribute to the low ADC values and the latter to the ill-defined margin on MR images. **g** Microscopy of a high-power field in a central area of the tumor shows a proliferation of tumor cells with atypical nuclei of various sizes. **h** In a peripheral area of the tumor, microscopy of a high-power field shows an infiltration of numerous inflammatory cells (arrows) in addition to the infiltrative growth of tumor cells

**Fig. 4:**
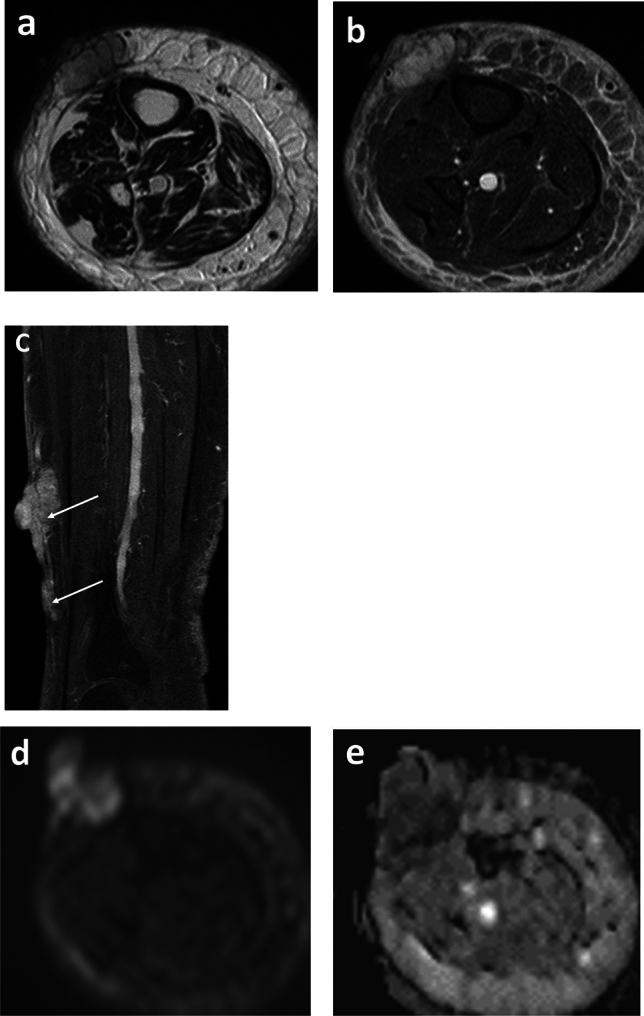
A 64-year-old female with an 8-year history of lower extremity disuse edema caused by cerebral infarction, presented with hemorrhagic masses of the left lower leg (case 2). **a** and **b** Axial T2-weighted and fat-suppressed T2-weighted images show an ill-defined mass with a confluent multinodular shape. **c** On sagittal fat-suppressed T2-weighted image, multiple masses in close proximity are identified (arrows). **d** and **e** DW image and ADC map show a hyperintense mass with low ADC values (ADC_mean_ = 0.83 × 10^−3^ mm^2^/s).

**Fig. 5:**
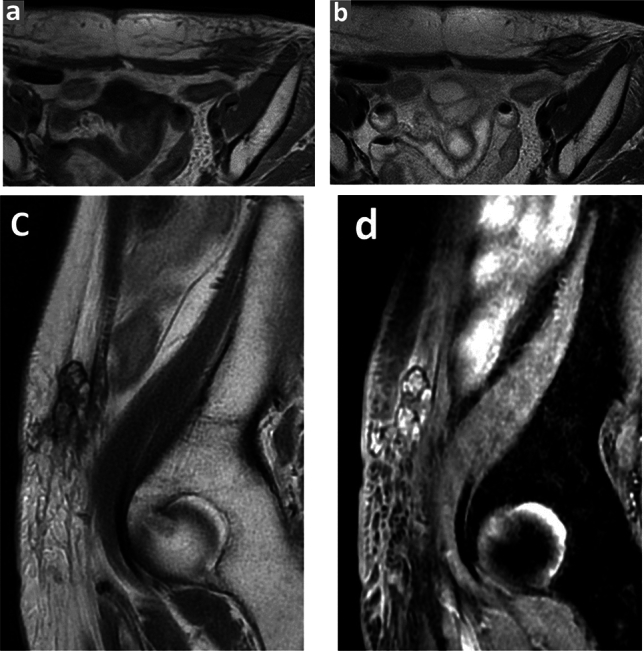
A 77-year-old female with a 10-year history of lymphedema following radical hysterectomy with pelvic lymphadenectomy for cervical cancer, presented with a palpable mass of the left lower abdominal wall (case 5). **a** and **b** Axial T1 and T2-weighted images show an ill-defined subcutaneous mass in the lower abdominal wall. **c** and **d** Sagittal T2- and fat-suppressed T1-weighted images show a confluent multinodular shaped mass with a mixture of intermediate and high signal intensities

The MRI and pathologic features are summarized in Table 2, and representative cases are presented in Figs. 3, 4, and 5. The tumor depth was the subcutis in three cases and the cutis and subcutis in two. Outer protrusion from the skin surface was observed in three cases. Four cases had multiple masses and one single mass. The mean maximal diameter of the largest lesion in each case was 6.0 cm, ranging from 4.3 to 10.4 cm. The tumor shape was confluent multinodular in three cases and amorphous or diffuse in two. The tumor margins were ill-defined in all cases. On T1-weighted images, two tumors showed a mixture of high and intermediate signal intensities with a predominance of intermediate signal intensity, and the remaining two showed intermediate signal intensity. On T2-weighted images, four tumors showed a mixture of high and intermediate signal intensities and one showed a mixture of intermediate and low signal intensities. Among these mixed signal intensities, the intermediate signal intensity was predominant in four cases. Our first presentation of ADC mapping was obtained in four cases. The mean ADC_mean_ value were 0.88 × 10^−3^ mm^2^/s, ranging from 0.77 × 10^−3^ to 0.96 × 10^−3^ mm^2^/s.

**Table 2:**
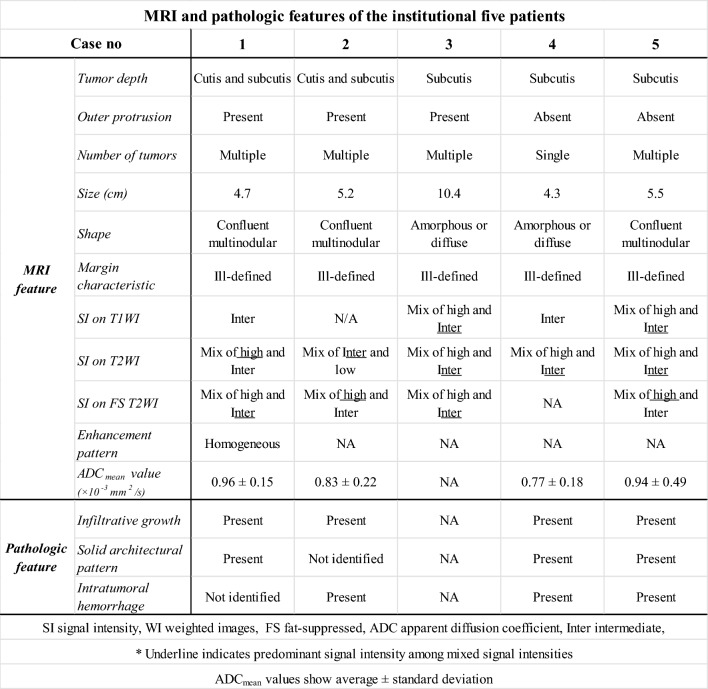
MRI and pathologic features of the institutional five patients

In the histopathologic correlations available for four cases, infiltrative growth of tumor cells with inflammatory cells was observed in all cases. In the central part of the tumor, a solid architectural pattern consisting of dense cell proliferation with relatively little stroma was observed in three cases. Intratumoral hemorrhage was identified in three cases.

### Article selection

The initial literature search yielded 123 articles, and after the removal of duplicated articles and screening of abstracts, 53 potentially eligible articles were assessed for further full-text review. After excluding 39 articles, 24 cases from 14 articles met the selection criteria for the systematic review [2, 7, 11–22]. A citation review identified an additional case from an article that met the selection criteria [23]. Finally, 25 cases from 15 articles were included in the systematic review (Fig. 1). Combined with five cases from our institutions, the final study cohort included 30 STS cases.

### Clinical features of the combined data

Table 3 summarizes the clinical features of the combined 30 patients. The patients included 25 females and 5 males, with a mean age of 60 years, respectively, ranging from 15 to 90 years. The most common cause of lymphedema was surgical intervention for breast cancer in 13 patients (13/23, 56.5%), followed by surgical intervention for uterine cervical cancer in four patients (4/23, 17.4%). The mean duration of lymphedema was 12 years, ranging from 8 to 20 years. The most common presenting symptom was a palpable mass (11/13, 84.6%), followed by skin discoloration or exanthesis (8/13, 61.5%). Except for two cases of lower abdominal origin (2/30, 6.7%), all tumors were located in the extremities; the most common sites were the lower leg (8/30, 26.7%) and forearm (8/30, 26.7%), followed by the upper arm (4/30, 13.3%), foot (4/30, 13.3%), thigh (3/30, 10.0%), and hand (1/30, 3.3%).

**Table 3:**
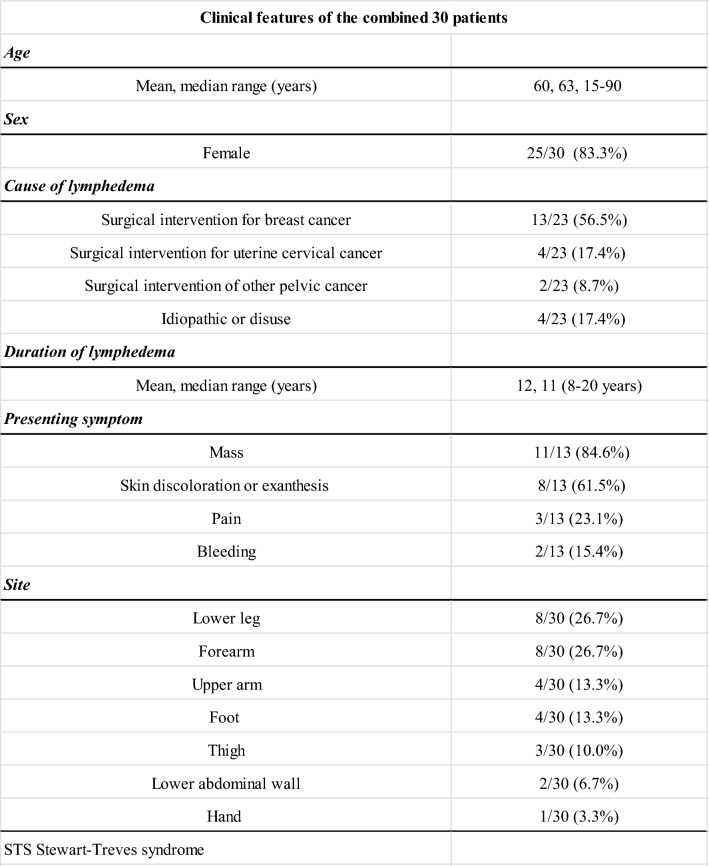
Clinical features of the combined 30 patients

### MRI features of the combined data

Table 4 summarizes the MRI features of the combined 30 patients. Tumor depth was at the cutis in two cases (2/25, 8.0%), cutis and subcutis in 17 (17/25, 68.0%), and subcutis in six (6/25, 24.0%). Ten of the 23 tumors involving the subcutis were in contact with the deep peripheral fascia; however, none extended beyond the fascia. Outer protrusions from the skin were observed in 10 tumors (10/22, 45.5%). Multiple tumors were observed in 16 cases (16/27, 59.3%). The tumor shape was round or oval in four cases (4/21, 19.0%), confluent multinodular in eight (8/21, 38.1%), and amorphous or diffuse in nine (9/21, 42.9%). The tumor margin was ill-defined in 26 cases (26/28, 92.9%). On T1-weighted images, the common signal intensity of the tumors was intermediate (16/19, 84.2%). In the remaining cases (3/19, 15.8%), the signal intensity was a mixture of intermediate and high, with an intermediate predominance. The signal intensities on T2-weighted images were intermediate in seven cases (7/13, 53.8%), a mixture of intermediate and high in five (5/13, 38.5%), and a mixture of intermediate and low in one (1/13, 7.7%). In all tumors with mixed signal intensities, the intermediate signal intensity was predominant. On fat-suppressed T2-weighted images, the common signal intensity was a mixture of intermediate (7/11, 63.6%). Contrast-enhanced images showed homogeneous enhancement in nine cases (9/12, 75.0%) and heterogeneous enhancement in three (3/12, 25.0%). In conclusion, angiosarcomas presenting as Stewart-Treves syndrome usually involved the subcutis and more than half were multiple. In more than 90% of cases, the tumor has an ill-defined margin and predominantly intermediate signal intensities on both T1- and T2-weighted images.

**Table 4:**
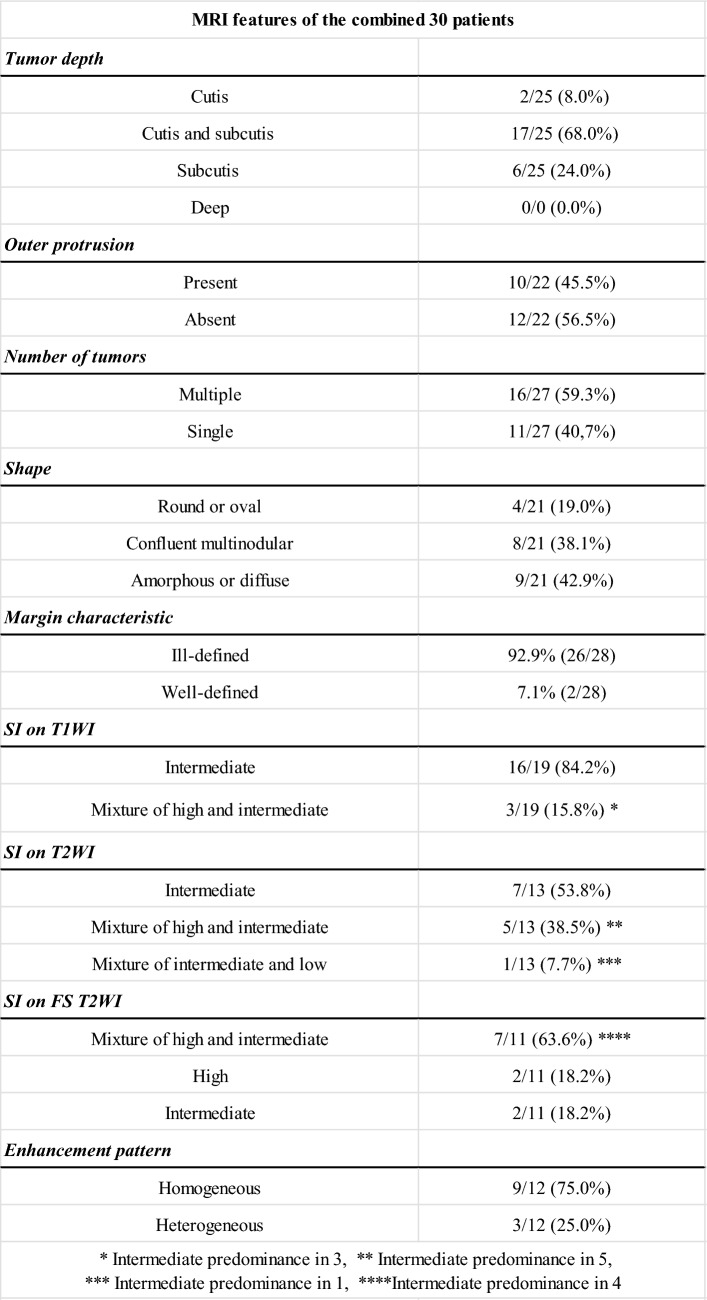
MRI features of the combined 30 patients

## Discussion

In this study, we first comprehensively summarized the MRI features of STS based on five institutional cases and 25 published cases, which showed the following typical features: both cutaneous and subcutaneous tumor depth, multifocal presentation, ill-defined tumor margin, and predominant intermediate signal intensities on both T1- and T2-weighted images.

In line with angiosarcomas of the scalp, which are the most frequent cutaneous angiosarcomas, the combined data showed that angiosarcomas in STS most frequently involved both the cutis and subcutis [24]. However, unlike scalp angiosarcomas, angiosarcomas in STS are occasionally confined to the subcutis. In addition, an outer protrusion from the cutis, which was found in almost all scalp angiosarcomas, was found in less than 50% of angiosarcomas in STS. This tendency for angiosarcomas in STS to occur more deeply may reflect a difference in etiology; that of angiosarcomas in STS is lymphedema, whereas that of scalp angiosarcomas is thought to be sun exposure [25, 26].

The multifocal presentation observed on MRI is consistent with reported pathologic features [3, 27] and may be related to the tumor shape; the tumor shapes varied and showed no particular trend. However, the most commonly observed confluent multinodular shape is thought to be the fusion of multiple round or oval masses into a single mass, which may reflect a tendency toward a multifocal presentation.

Previous MRI studies of soft tissue tumors have reported that an ill-defined tumor margin is an overlapping finding that can be seen in both benign and malignant soft tissue tumors [28, 29] but is a suggested predictor of high-grade tumors within soft tissue sarcomas [30, 31]. Although angiosarcomas were not included in these previous studies, our data on the margin characteristics were consistent with those of other high-grade soft tissue sarcomas. Regarding pathologic features corresponding to the ill-defined margin on MR images, infiltrative growth of tumor cells has been reported as a common pathologic　feature of angiosarcomas in STS [3, 7], and this was observed in our institutional cases. In addition, the pathological features in our cases showed infiltration of reactive inflammatory cells, which is also thought to contribute to the ill-defined margins on the MRI images.

The internal signal intensities of the tumors on conventional MR sequences also showed a constant trend, with predominantly intermediate signal intensities on both T1- and T2-weighted images. This finding is also nonspecific because a variety of benign and malignant soft tissue tumors show similar signal intensities, even when localized to superficial soft tissue masses. However, intermediate signal intensities may be helpful in narrowing the differential diagnosis, for example, to exclude ganglia, myxoid tumors, and abscesses [28, 29, 32]. Regarding the hyperintense area on T1-weighted images, we identified it in two of four our cases with available T1-weighted images, whereas we identified it in only one of 15 systemic review cases with available T1-weighted images. This discrepancy may be explained by the fact that the majority of articles from the systematic review did not focus primarily on MRI imaging. Since hemorrhage has been reported to be a common pathological feature of angiosarcomas [3, 6, 7], it will be a future issue to evaluate MRI findings reflecting hemorrhage including hyperintense area on T1-weighted images in a larger group of patients.

Our institutional cases first presented DW images of angiosarcomas in STS, with ADC_mean_ values of less than 1.00 × 10^−3^ mm^2^/s in all four cases. These low ADC_mean_ values were suggested to reflect the predominance of solid architectural patterns composed of dense cellular sheets by histopathologic correlation and were consistent with the ADC values of other non-myxoid malignant soft tissue tumors [33, 34]. Although this assumption was based on a limited number of cases, a low ADC value may aid in the diagnosis of STS.

This study had several limitations. First, the number of institutional cases was small, although the data were collected from three cancer referral centers. Secondly, the heterogeneity of the collected studies resulted in missing clinical and imaging data. Third, the preponderance of case reports in our review may have resulted in publication bias. Fourth there was no comparison with other tumors that can occur in chronic lymphoedema because other tumors are extremely rare. However, owing to the rarity of this condition, it may be difficult to collect data from uniform MRI sequences in a larger cohort of patients. Therefore, the comprehensive MRI features presented herein can be considered significant.

In conclusion, STS should be suspected in patients with chronic lymphedema who develop multiple superficially located masses with ill-defined margins. The intermediate signal intensities of the mass on both T1- and T2-weighted images and the low ADC values of the mass may aid in the diagnosis of this rare entity.
